# Supplementary material for: In Vitro Antibacterial Activity of Ethanolic Extracts Obtained from Plants Grown in Tolima, Colombia, Against Bacteria Associated with Bovine Mastitis
Source: Vet Sci. 2025 Sep 18;12(9):903. doi: 10.3390/vetsci12090903 (PMC12474195; doi:10.3390/vetsci12090903)
Supplement: Supplementary file 1 [file vetsci-12-00903-s001.zip › vetsci-3690022-supplementary.pdf]

Supplementary material

Figure S1. Inhibition zones of ethanolic extracts: *M. chamomilla*, *C. officinalis*, *R. officinalis*, *P. guajava* and *C. papaya*. Positive control (C⁺) (Erythromycin 10 mg/mL). C*oagulase positive Staphylococcus* **(A)** and *Streptococcus spp* **(B)**.

(A) C*oagulase positive Staphylococcus*

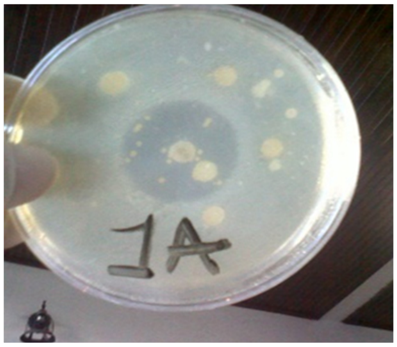

M. chamomilla

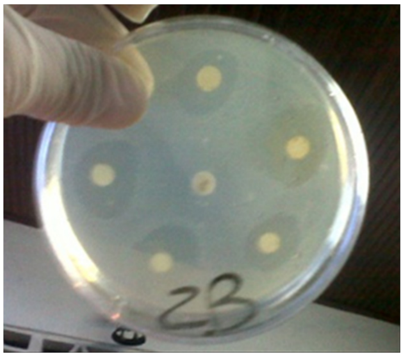

C. officinalis

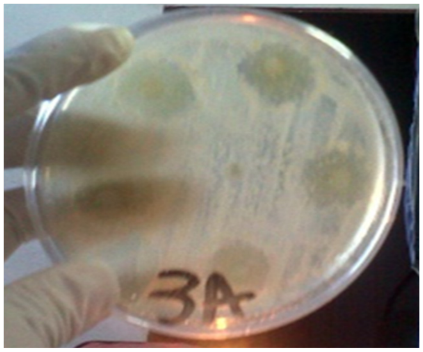

R. officinalis

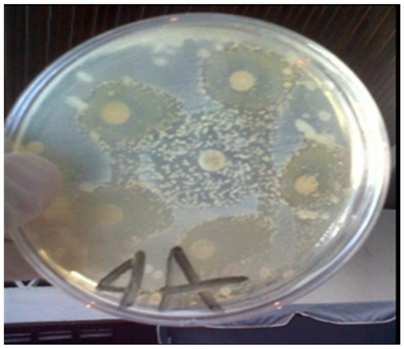

P. guajava

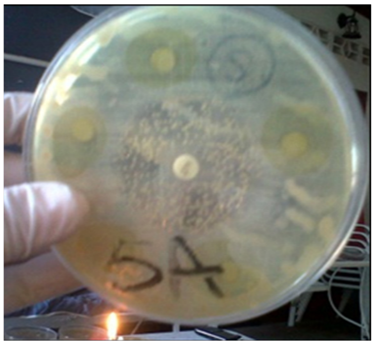

C. Papaya

(A) *Streptococcus spp*

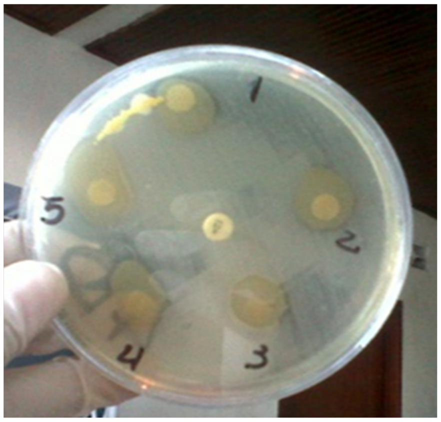

M. chamomilla

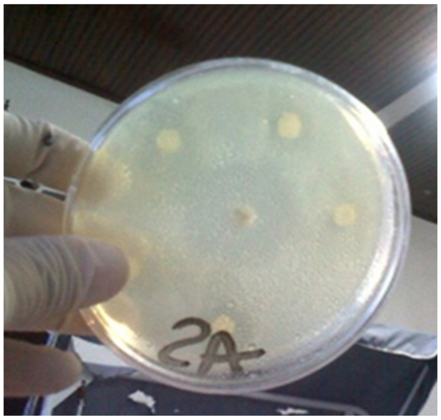

C. officinalis

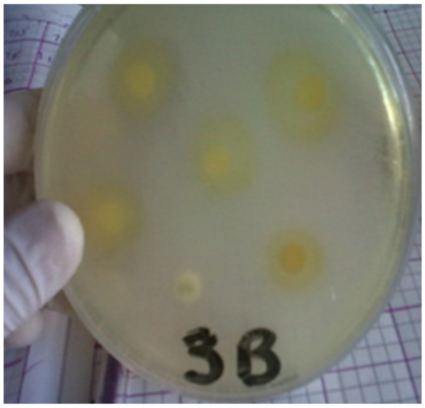

R. officinalis

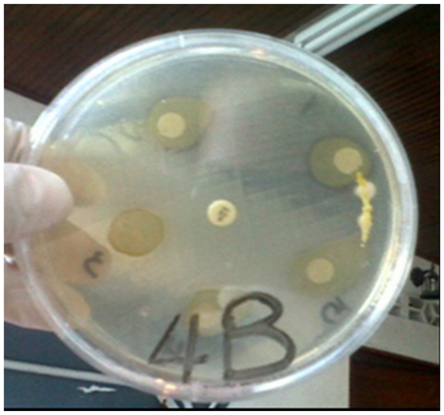

P. guajava

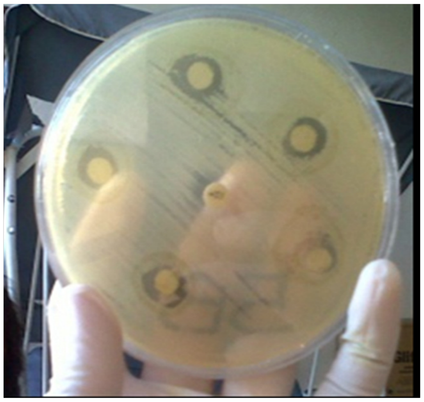

C. Papaya

Table S1. Amount of extract, solvent, solvent extract ratio, and yield percentage

Plant species name	Ground plant material (g)	Solvent (mL)	Relation Ground plant material- Solvent	Dry extract weight (g)	yield (%)
Calendula. officinalis L	150	450	1:3	21.0	14.0
Psidium guajava L	150	450	1:3	22.0	14.6
Matricaria. chamomilla L	150	450	1:3	12.5	8.3
Rosmarinus officinalis L	150	450	1:3	7.9	5.3
Carica papaya L	150	450	1:3	13.6	9.1
